# A Systematic Review of the Effect of Expectancy on Treatment Responses to Acupuncture

**DOI:** 10.1155/2012/857804

**Published:** 2011-11-14

**Authors:** Ben Colagiuri, Caroline A. Smith

**Affiliations:** ^1^Centre for Complementary Medicine Research, University of Western Sydney, NSW 2751, Australia; ^2^School of Psychology, University of New South Wales, Kensington, NSW 2052, Australia

## Abstract

Randomised controlled trials (RCTs) of acupuncture often find equivalent responses to real and placebo acupuncture despite both appearing superior to no treatment. This raises questions regarding the mechanisms of acupuncture, especially the contribution of patient expectancies. We systematically reviewed previous research assessing the relationship between expectancy and treatment responses following acupuncture, whether real or placebo. To be included, studies needed to assess and/or manipulate expectancies about acupuncture and relate these to at least one health-relevant outcome. Nine such independent studies were identified through systematic searches of Medline, PsycInfo, PubMed, and Cochrane Clinical Trials Register. The methodology and reporting of these studies were quite heterogeneous, meaning that meta-analysis was not possible. A descriptive review revealed that five studies found statistically significant effects of expectancy on a least one outcome, with three also finding evidence suggestive of an interaction between expectancy and type of acupuncture (real or placebo). While there were some trends in significant effects in terms of study characteristics, their generality is limited by the heterogeneity of study designs. The differences in design across studies highlight some important methodological considerations for future research in this area, particularly regarding whether to assess or manipulate expectancies and how best to assess expectancies.

## 1. Introduction

Many studies comparing real acupuncture to placebo controls fail to find statistically significant differences between these two treatments but often find that both real acupuncture and the placebo controls produce better outcomes than no treatment or standard care alone [1–4]. This suggests that there is some benefit to providing acupuncture treatment, whether real or placebo, but raises questions about the underlying mechanisms of these effects. The three most common explanations proposed to account for improvements following both real and placebo acupuncture are that (1) needling is only one of a variety of active components in acupuncture treatment, (2) the placebo controls used in the studies are, in fact, active treatments and, therefore, invalid placebos, or (3) improvement following both real and placebo acupuncture results from the placebo effect.

Placebo (or sham) control in randomised placebo-controlled trials (RCTs) involves comparing the therapy of interest with a dummy treatment so that all participants engage in a treatment process, but only those allocated to the target therapy receive the specific component being tested [5]. Acupuncture is a complex intervention involving diagnosis, needling, facilitating patients active involvement in their recovery, lifestyle advice, and therapeutic alliance, all of which are tailored individually to the patient being treated [6]. Some researchers have argued that these components cannot be validly partitioned and that assessing individual components will underestimate the true efficacy of acupuncture, because the response to the whole acupuncture intervention may be greater than the sum of responses to the components of acupuncture administered individually [6–10]. If so, this means that RCTs, which seek to isolate and test the efficacy of a single component, may not be appropriate for assessing acupuncture. This would suggest that a lack of difference between real and placebo acupuncture in RCTs may result from the omission of important components of acupuncture, such as facilitating patients active involvement in their recovery and lifestyle advice, that is common in these trials [6, 11]. However, before such a conclusion can be drawn, evidence is required that demonstrates a larger benefit of providing acupuncture treatment than summing the benefit of providing the individual components of acupuncture alone, which, to our knowledge, has not yet been tested.

Placebo (or sham) controls adopted in RCTs of acupuncture include needle insertion at nonacupuncture points (sham acupuncture), shallow needle insertion that does not penetrate below the skin (minimal or superficial needling), and blunt needles that touch, but do not penetrate the skin (placebo needling). Lundeberg and colleagues [12–14] have argued that these techniques are not inert and are, therefore, invalid as placebo controls. They provide a list of eleven reasons why the placebo controls used in acupuncture RCTs may be active treatments, including evidence of physiological responses to sham acupuncture, evidence that superficial and sham needling producing larger effects than a placebo pill, and, rather strangely, that placebo controls can be as effective or even more effective than real acupuncture. 

However, the evidence provided by Lundeberg et al. [14] can be explained equally well in the context of patient expectancies. Expectancy is proposed to be a key mechanism of the placebo effect. Placebo effects are changes that occur in response to receiving treatment but that are not due to the inherent properties of the treatment itself [15]. Many studies have found that a saline injection or placebo cream administered under the guise of a powerful analgesic can, in fact, reduce pain, for example [16–22]. There is also evidence for placebo effects across a range of other conditions (see [23] for a recent review). For example, placebo treatment appears to reduce depressive symptoms [24], improve sleep quality [25] improve motor performance in patients with Parkinson's disease [17], modulate heat rate in healthy volunteers [17], and improve cognitive performance in healthy volunteers [26]. Perhaps most interestingly, Benedetti et al. [27] found significantly larger treatment effects for postoperative pain, motor performance in patients with Parkinson's disease, and heart rate in healthy participants when the initiation of treatment was signalled to the patient by a health professional compared with when it was initiated surreptitiously without the patients' awareness, indicating that most medical treatments involve a placebo component. On this basis, some researchers have argued that the superiority of both real and placebo acupuncture techniques over no treatment (or in some cases standard care) combined with failure to find significant differences between real and placebo acupuncture can be explained by the placebo effect [28, 29]. That is, they argue that any improvement following acupuncture treatment, whether real or placebo, results from the patients expecting acupuncture to be effective. 

If expectancies do lead to real changes in symptoms via the placebo effect, then physiological changes must underlie these effects. Therefore, the physiological changes Lundeberg et al. [14] cite following placebo acupuncture do not discount the possibility of expectancy effects. There is also evidence that the more invasive the placebo, the larger the placebo effect. For example, four placebo pills reduced recovery times from duodenal ulcers compared with two placebo pills [30] and a subcutaneous placebo injection reduced pain due to migraine headaches more effectively than a placebo pill [31]. As such, placebo acupuncture may simply produce stronger expectancy effects than placebo pills do. Finally, if both real and placebo acupuncture exert their effects as a result of expectancy, then this would lead to frequent null differences and occasional statistically significant differences between the two treatments caused by sampling variation (cf. Type I error [32]), including placebo acupuncture appearing superior to real acupuncture on occasion As a result, there is as yet no conclusive evidence that the currently used placebo controls are active beyond expectancy. 

Perhaps more importantly, the three alternative explanations for the common lack of statistically significant differences between real and placebo acupuncture are not mutually exclusive. Needling may be more efficacious when delivered with lifestyle advice, but this does not mean that patients' expectancies about the efficacy of an acupuncture intervention cannot influence their outcomes via the placebo effect. Similarly, currently used placebo controls for acupuncture needling could be invalid, but this does not preclude the possibility that expectancies could contribute to responses to real acupuncture. As demonstrated by Benedetti et al. [27], most medical treatments, whether efficacious or not, appear to be influenced by patient expectancies. Thus, regardless of whether or not the combined effects of an acupuncture intervention cannot be explained by the effects of each component's individual efficacy or whether or not the currently used placebo controls in acupuncture RCTs are valid, it remains important to establish both if and how the placebo effect contributes to responses to acupuncture. 

With this in mind, we conducted a systematic review of the literature to examine whether expectancies can influence acupuncture outcomes. Although we had intended to use meta-analysis to estimate and test the magnitude of the effect of expectancy on treatment responses following acupuncture, the studies identified were too heterogeneous with respect to methodology and reporting to allow such analysis. We, therefore, provide a descriptive review of studies investigating placebo effects in acupuncture, drawing particular attention to methodological considerations, and outline some key goals for future research in this area.

## 2. Methods

### 2.1. Search Strategy

Articles were identified through computerized literature searches. Medline, PsycInfo, PubMed, and Cochrane Clinical Trials Register were searched for English publications from inception up to 1st December, 2010 using the search terms “expectancy OR expectancies OR expectation$ OR expected efficacy OR placebo effect$” in combination with “acupuncture” using title and abstract fields. The reference lists of publications identified through the electronic search were also screened for additional relevant articles. 

### 2.2. Selection Criteria

To be included, studies needed to either assess or manipulate participants' expectancies regarding the efficacy of an acupuncture intervention involving needling and to report on the relationship between these expectancies or the manipulation and at least one outcome variable. The acupuncture intervention could include manual or electroacupuncture and could be standardised or individualised. Assessing expectancies regarding the efficacy of acupuncture involved any question asking participants to rate their expectancies for improvement as a result of acupuncture but had to be prospective; that is, the expectancy assessment had to occur before the acupuncture treatment. Manipulating expectancies meant allocating participants to receive different information about the likely effects of their treatment, whether real or placebo acupuncture was delivered. For example, Suarez-Almazor et al. [33] randomly allocated participants in a RCT comparing real and sham acupuncture for osteoarthritis of the knee to receive suggestion from the acupuncturist that either the treatment “will work” (high expectancy) or that it “may or may not work” (low expectancy). Studies investigating both clinical and nonclinical conditions (e.g., experimentally-induced pain) were included. The studies could assess any health-related outcome, whether subjective or objective, and there were no constraints on study design, as long as the criteria for assessing and/or manipulating expectancies were met. Only peer-reviewed publications in English were included. 

### 2.3. Study Selection

One author (B. Colagiuri) conducted the initial search and excluded articles that were clearly not relevant. Both authors then reviewed the full texts of each of the remaining articles and evaluated them against the selection criteria independently. Any disagreements were resolved through discussion. 

**Figure 1:**
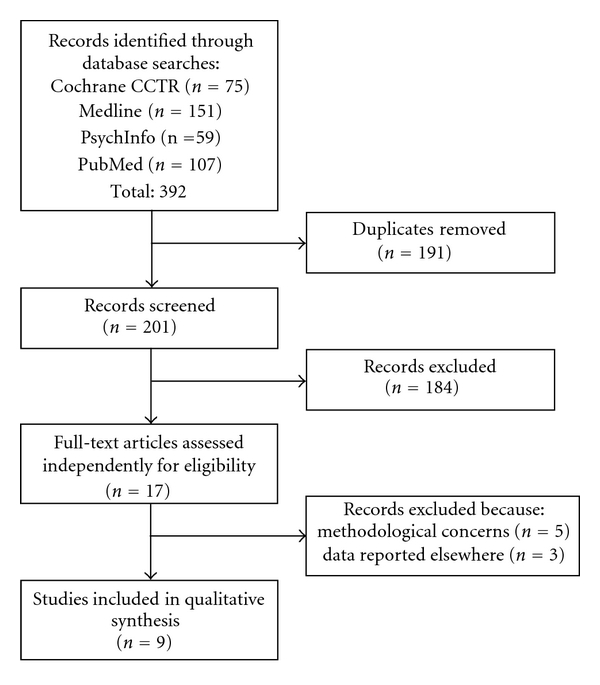
Flow diagram for study identification and selection.

The literature search identified a total of nine independent studies reporting on the relationship between expectancy and treatment response following acupuncture suitable for inclusion.  Figure 1 displays the flow diagram for study selection. The search of Medline, PsycInfo, PubMed, and Cochrane Clinical Trials Register provided a total of 392 English references. After removing duplicates, there were 201 articles, of which 184 were clearly not relevant. The full texts of the remaining 17 articles were reviewed independently by both authors. Of these, three articles were excluded because their results were reported in other articles already identified [34–36]. This left 14 unique studies. One article was excluded because it reported on the relationship between expectancy and acupuncture combined with expectancy and an exercise intervention [37]. One article was excluded because no details of the expectancy assessment were provided [38]. One was excluded because it focused on patients with psychological comorbidity [39], which although not an *a priori *exclusion criteria, both authors agreed might affect the relationship between expectancy and treatment outcomes. One was excluded because it only assessed participants' expectancies retrospectively in the form of guesses about treatment allocation [29]. One was excluded because it failed to directly test the effect of its expectancy manipulation [40].

### 2.4. Data Extraction

The authors reviewed the retrieved articles and independently extracted information on sample characteristics, study design, outcome variables, relevant results, and whether the study fulfilled the inclusion criteria using pre-defined coding sheets. The sample characteristics included sample size, proportion of female participants, and whether the participants had previously used acupuncture. Study design included the experimental design, characteristics of the acupuncture treatment that was delivered, and how expectancies were either assessed or manipulated. Study outcomes involved all outcomes that were analysed for relationships with expectancy and were classified into either self-report or objective outcomes. Differences were discussed, and a final assessment was negotiated for each study. The PRISMA guidelines for reporting of systematic reviews and meta-analyses were followed [41, 42]. 

### 2.5. Risk of Bias Assessment

Scoring studies numerically based on their quality is controversial. This is because combining quality items into a single score is questionable, particularly in terms of whether or not these items are additive [43, 44], and because there is evidence that currently used quality scores do not actually predict variance in effect sizes [45, 46]. We, therefore, chose not to attribute quality scores to the included studies. Instead, we conducted a risk of bias assessment using the Cochrane Collaborations tool for assessing risk of bias [47], which includes six dimensions, namely, adequate sequence generation, allocation concealment, blinding, incomplete data, selective reporting, and other forms of bias. Both authors completed the risk of bias assessment for each study independently, with any discrepancies resolved through discussion.

### 2.6. Data Analysis

Meta-analysis of the studies was not possible due a combination of heterogeneous methodology used across studies and incomplete reporting of results in some studies. Study results were considered statistically significant if *P* < 0.05. 

## 3. Results

### 3.1. Study Characteristics

A summary of the characteristics of the nine studies we identified is provided in Table 1. The majority of studies were on pain-related conditions, both clinical [33, 48–51] and experimentally-induced [52–54]. One study focused on angina pectoris [55]. In six of the studies, participants were acupuncture naive [33, 48, 51–54], in two studies, participants had not previously received acupuncture for the condition being treated [50, 55], and in one study no information was provided on participants' previous use of acupuncture [49]. Electro acupuncture was used in five studies [33, 52–55], manual acupuncture was used in three studies [48, 49, 51], and one study only investigated placebo acupuncture [50]. Five of the studies assessed expectancies [49–51, 53, 55], four manipulated expectancies [33, 48, 52, 54]. Assessing expectancies generally involved asking participants to rate how effective they expected acupuncture to be for improving their condition on Likert-type scales. In the majority of studies assessing expectancies, participants were either dichotomised into high and low expectancies [49, 53, 55] or trichotomised into high, medium, or low expectancies [51]. Manipulating expectancies typically involved randomising participants to receive information aimed at enhancing their expectancies for improvement following acupuncture or either neutral or negative information although one study used a conditioning procedure [54]. All studies included self-reported outcomes, but three also included objective outcome variables [33, 48, 55]. 

### 3.2. The Effect of Expectancy on Responses to Acupuncture

Table 2 provides a descriptive summary of each of the nine studies' findings. The results of the studies were clearly mixed, with some studies finding at least some evidence of a statistically significant effect of expectancy on acupuncture outcomes [33, 49, 52–54] and others failing to find any such effects [48, 50, 51, 55]. Interestingly, there were also some findings that were suggestive of an interaction between expectancy and type of acupuncture (real versus placebo). For example, Linde et al. [49] found that the improvement in patients classified as having “high expectancy” compared with those classified as having “low expectancy” was significantly more marked in patients receiving real acupuncture compared with placebo acupuncture. However, evidence of this type of interaction was inconsistent across the studies with some studies finding evidence suggestive of an interaction [49, 52, 53] and others failing to find such evidence [33, 54]. Interaction effects were either not reported [48, 51, 55] or not relevant (because only one acupuncture treatment was administered [50]) in the remaining studies. No study found evidence of significant effects of expectancy on objective outcomes following acupuncture; however, only three studies included objective outcome variables [33, 48, 55]. 

There were some patterns in terms of the study characteristics and whether or not a significant relationship between expectancy and acupuncture outcomes was found. All three studies investigating experimentally-induced pain found evidence of a significant relationship [52–54], whereas only two of the six studies investigating clinical outcomes found evidence of a significant relationship [49, 51]. Three of the four studies that manipulated expectancies found evidence of a significant relationship [33, 52, 54], whereas only one of the five studies that assessed expectancies found evidence of a significant relationship [49]. Four of the five studies involving electroacupuncture found evidence of a significant relationship between expectancies and treatment response [33, 52–54], whereas only one out of the four studies involving manual acupuncture found evidence of such a relationship [49]. A high degree of caution is, however, necessary when attempting to generalise from these patterns as simple vote counting, that is, summing and comparing the number of significant results with the number of nonsignificant results, is associated with a number of problems [56]. In the current case, for example, even though only two of the six studies investigating clinical outcomes found evidence of a significant relationship between expectancy and acupuncture outcomes [33, 49], these were the two largest in terms of sample size and likely had the most statistical power. The same applies to the only study finding a significant relationship that assessed expectancies [49]. It is also worth noting that studies with healthy volunteers in experimental settings should require fewer participants to achieve the same power as studies in clinical settings, because the former are often better able control for potential confounding variables due to the controlled laboratory setting, which further complicates comparison across these studies. Therefore, while it seems clear that expectancies can affect acupuncture outcomes under at least some circumstances, it is difficult to identify which circumstances these are and how strong this relationship is from the available evidence. 

### 3.3. Risk of Bias

As shown in Table 3, all but one study [33] had either some risk or an unclear risk of bias on at least one of the six dimensions assessed. Specifically, sequence generation was inadequate in one study [52] and unclear in four studies [48, 53–55]. Allocation concealment was not used in one study [52] and was unclear in three studies [48, 54, 55]. Participants were blinded to whether or not they were receiving real or placebo acupuncture in all studies, but in four studies the blinding of outcome assessors was unclear [48, 49, 53, 54]. All studies satisfactorily addressed incomplete data, and only one had unclear risk regarding selective reporting [55]. In terms of other biases, four studies simplified their expectancy assessment via dichotomisation or trichotomisation and three studies [49, 51, 53, 55] had relatively small sample sizes given their correlational nature [50, 53, 55].

## 4. Discussion

Given that patient expectancies are often proposed to be a key factor in acupuncture's effectiveness compared with no treatment or standard care [28, 29], relatively few studies have examined the relationship between expectancies and treatment responses following acupuncture. Our systematic search identified only 14 unique studies testing the relationship between patient expectancies and outcomes following acupuncture needling, of which nine met our criteria for inclusion. The high level of heterogeneity across studies and incomplete reporting in some meant that meta-analysis was not possible. A descriptive review revealed that while there was evidence of a significant relationship between patient expectancies and acupuncture needling outcomes in some studies, others failed to find these effects. The pattern of results suggested that studies on experimentally-induced pain, that manipulated expectancies, or those involving electroacupuncture were more likely to find a significant relationship. However, caution is required in generalising these results, as it was more common for studies on experimentally-induced pain to manipulate expectancies and to employ electro-acupuncture, meaning that the effects of each cannot be disentangled on the basis of the available data. Further, the largest study on a clinical outcome, that assessed expectancies, and that involved manual acupuncture, did find evidence of a significant relationship between expectancy and acupuncture outcomes [49]. It was also the case that some studies were at higher risk of bias than others. 

The differences in study design and inconsistent results across the identified studies raise important considerations regarding which methodological approach is best equipped to determine the contribution of patient expectancies to acupuncture outcomes. The two most pertinent methodological issues are (1) whether to assess or manipulate expectancies and (2) how to accurately assess expectancies. 

Of the nine studies identified here, five assessed expectancies [49–51, 53, 55] and four manipulated expectancies [33, 48, 52, 54]. Studies that involve manipulating expectancies are better able to determine how patient expectancies contribute to acupuncture outcomes because of their experimental nature and might be considered superior for this reason. However, studies that only manipulate expectancies are entirely reliant on the ability of the manipulation to influence expectancies. This leads to problems determining whether an unsuccessful manipulation failed because it did not sufficiently influence expectancies or because the participants' expectancies had no effect on their treatment response, as is the case in Berk et al.'s [48] study. Studies that assess expectancies have the advantage of being able to directly evaluate the relationship between expectancy and acupuncture outcomes, thereby overcoming problems to do with relying on the efficacy of an expectancy manipulation. However, these types of studies might be considered a weaker source of evidence because they are correlational in nature. 

An apparently simple way to overcome this issue is to include an assessment of expectancy in studies involving manipulations. However, there are a number of other potential limitations associated with assessing expectancies that need consideration. First, questioning participants about their expectancies regarding acupuncture's efficacy could undermine the study's validity if it influences what they expect or if it makes them question the purpose of the study. Second, determining the best time to assess expectancies is also difficult. Assessing them immediately before the first acupuncture treatment provides a prospective assessment, but expectancies may change during the course of the treatment, especially if it lasts for more than a few days. On the other hand, assessing expectancies immediately before or immediately after the outcomes are assessed could lead to priming that artificially inflates the strength of the relationship between expectancy and the outcome. Thirdly, there have been few systematic attempts to develop methods of assessing expectancies, both within acupuncture research and in the placebo literature more broadly. Most of the studies that assessed expectancies identified here used a single expectancy item. For the most part, these were 5-point Likert-type scales, although, as can be seen in Table 1, both the wording of the question and the labels for the response options varied considerably. It was also common for studies assessing expectancies to dichotomise [49, 53, 55], or in one case trichotomise [51], patients' responses into different levels of expectancy, however, categorising such variables has been heavily criticised, because it can substantially reduce statistical power [57–59]. 

Therefore, while studies that both manipulate and assess expectancies are best able to test the relationship between expectancy and acupuncture outcomes, questions regarding the influence of asking patients to report their expectancies and both when and how expectancies should be assessed need to be addressed empirically in order to determine the most appropriate method of assessing expectancies. Of course, it may not always be practical to incorporate an expectancy manipulation into a trial of acupuncture, as this may require substantially larger samples to achieve the same level of power or may raise ethical considerations if deception is required. In these circumstances, it is still useful to assess expectancies as this can provide estimates of the relationship between expectancy and treatment responses following acupuncture, but again, the best methods of assessing expectancy need to be tested empirically in order to maximise the validity of such research.

There are three potential limitations to the current review. Firstly, as noted above, we were unable to conduct meta-analysis to estimate and test the effect size for the relationship between expectancy and acupuncture outcomes due to the high heterogeneity in methodology and incomplete reporting in some studies. While this does mean that we were unable to determine an average effect size across studies, the descriptive review provided here does highlight a number of important methodological considerations that will inform future research in this area. Secondly, as with most systematic reviews, there is the possibility of publication bias. In the current case, this could mean that studies failing to find a statistically significant relationship between expectancy and acupuncture outcomes were less likely to be published than those finding statistically significant effects, which may lead to overestimation of the influence of expectancy. We, therefore, encourage researchers conducting RCTs of acupuncture to report, even briefly, of any failures to find a significant relationship between expectancy and acupuncture outcomes. Finally, only papers published in English were reviewed, meaning that other relevant studies may be published in other languages. 

In summary, there have been relatively few research studies testing the relationship between expectancy and acupuncture outcomes. While there did appear to be evidence for a significant relationship between patient expectancies and treatment responses following acupuncture, there were some inconsistencies across studies. Future studies attempting to address this question should, where possible, both manipulate and assess expectancies. However, considerations regarding currently used methods of assessing expectancy, such as timing and wording of the questions, need to be addressed first in order to establish the best approach and to ensure the validity of these assessments and any conclusions drawn about the relationship between expectancy and acupuncture outcomes. Further, investigating potential moderators of the relationship between expectancy and acupuncture outcomes, such as type of acupuncture (real versus placebo), type of stimulation (manual versus electroacupuncture) would prove useful for better understanding the circumstances under which expectancies can influence treatment responses following acupuncture. 

## Figures and Tables

**Table 1:** Summary of included studies' characteristics.

Study	Design		Sample		Treatment		Expectancy	Outcome
		*N*	% Female	Previous use	Acupuncture^a^	Placebo		
Berk et al. (1977) [48]	2 × 2 between-subjects design with acupuncture (real versus placebo) and milieu (positive versus negative) as factors on shoulder pain.	42	29%	No	Acupuncture at LI11, LI4, LI15, GB39, SI9, S10, and M-UE-48 three times over 3 weeks. Needles were manually manipulated, but retention time was not reported.	Noninsertion at the study acupuncture points involving gently pressing the tip of the needle against the skin.	Manipulated—participants randomised to receive acupuncture with positive milieu suggesting that acupuncture is an effective therapy or a negative milieu suggesting that acupuncture is an ineffective treatment.	Objective—shoulder mobility. Self report—pain.

Knox et al. (1979) [52]	3 × 3 between-subjects design with acupuncture (real, placebo, or none) versus expectancy (positive, negative, or variable) for experimentally-induced pain (cold pressor).	72	50%	No	Electroacupuncture at LI4 and TH5 once for 20 min unilaterally on the arm to be placed in the cold pressor. Sensation not reported.	As per acupuncture, but stimulated study points unilaterally on the arm not placed in the cold pressor. A no treatment control group lay down for 20 min and did not receive either treatment.	Manipulated—participants led to expect pain relief, no pain relief, or variable effects from acupuncture or from lying down for 20 min.	Self report—pain at 30 sec.

Norton et al. (1984) [53]	RCT of acupuncture versus placebo for experimentally-induced pain (cold pressor).	24	50%	No	Electroacupuncture at LI5, LI11, SI5, and SI8 once for 15 min unilaterally on the arm to be placed in the cold pressor.	Insertion 2 cm distal to study acupuncture points.	Assessed—expectancy questionnaire comparing treatments (e.g., surgery, morphine, aspirin, and acupuncture) for relieving pain and then categorised participants in to high and low expectancy on the basis of this questionnaire.	Self Report—pain.

Ballegaard et al. (1995) [55]	RCT of acupuncture versus placebo for angina pectoris.	32	22%	Not for heart disease.	Electroacupuncture LI4 for 20 mim. Ten treatments over 3 weeks. De qi and visible muscle twitch achieved.	Superficial (shallow) insertion outside Chinese meridians and not on trigger points with no stimulation.	Assessed—rating of expectancy concerning anti-anginal effects of acupuncture as “very high expectations”, “somewhat high”, “neutral”, “slightly negative”, “moderately negative expectations”, or “don't know”. These scores were dichotomised into either maximal expectation consisting of those who responded “very high expectations” and into submaximal expectations for all others responses.	Objective—exercise tolerance; rate pressure product; nitroglycerin consumption; angina attack rate. Self report—daily wellbeing.

Linde et al. (2007) [49]	Pooled analysis of 4 RCTs of acupuncture versus placebo for migraine, headaches, back pain, and osteoarthritis of the knee.	864	75%	Not stated.	Acupuncture protocol specific to RCT, but all were treated once per week for 12 weeks and each session lasted 30 min.	Superficial needling at nonacupuncture points (relevant to each RCT) also once per week for 12 weeks and each session lasting 30 min.	Assessed—(a) “How effective do you consider acupuncture in general?” and could respond “very effective”, “effective”, “slightly effective”, “not effective”, or “don't know”. (b) “What do you personally expect from the acupuncture you will receive?” and could respond “cure”, “clear improvement”, “slight improvement”, “no improvement”, “don't know”. Dichotomised into high expectancy (top two responses) versus low expectancy (all other responses).	Self report—50% improvement in primary outcome related to trial condition; pain disability index.

Bertisch et al. (2009) [50]	Comparison of placebo acupuncture versus placebo pill within a larger RCT for distal upper arm pain due to RSI.	60	53%	Not for arm pain and not within last year.	N/A	Streitberger placebo needles twice per week for 2 weeks at between 5–10 sites and unilaterally or bilaterally depending on the patients pain.	Assessed—“rate how intense you think the pain or discomfort will be 2 weeks from now if you are assigned to acupuncture” 5-point scale.	Self report—pain.

Kong et al. (2009) [35, 54]	2 × 2 between-subjects design with acupuncture (real versus placebo) and expectancy (high versus low) as factors for experimentally-induced pain (heat stimulation).	48	50%	No	Electroacupuncture at LI3 and LI4 once for 25 min. Di qi achieved.	Streiberger placebo needles placed on the surface of the skin at the study acupuncture points and connected to a deactivated electroacupuncture device.	Manipulated—participants given stimulation of pain with intensity surreptitiously manipulated so as to provide experience of acupuncture treatment decreasing pain (high expectancy) or with intensity identical to baseline so as to provide experience of acupuncture failing to decrease pain (low expectancy).	Self report—pain.

Sherman et al. (2010) [51]	RCT of individualised acupuncture, standardised acupuncture, placebo acupuncture, and standard care for chronic back pain.	477	61%	No	(a) Individualised acupuncture with points and sensation determined based on patients' individual diagnosis. Ten treatments in 7 weeks. (b) Standardised acupuncture at B23, B40, K3 bilaterally and Du3, main trigger point unilaterally for 20 min with manual stimulation to elicit “de qi”.	(a) Placebo acupuncture involving sham insertion using a toothpick in a needle guide tube as per the standardised acupuncture, including manipulation via twisting the tooth pick. (b) Standard care was the usual care participants received from their physicians, if any.	Assessed—participants rated how helpful they believed acupuncture would be for their back pain on 11-point scale. Responses trichotomised into low (0–5), medium (6 and 7), and high (8–10).	Self report—disability; symptom bothersomeness.

Suarez-Almazor et al. (2010) [33]	2 × 2 trial with communication style (positive or negative) and acupuncture (real or placebo) as factors and an additional waitlist control group for osteoarthritis of the knee.	527	61%	No	Electro-acupuncture at GB34, SP6, SP9, Ear-Knee, Ex-LE2, Ex-LE4, Ex-LE5, and 1-2 trigger points. Needle retention was 20 min and treatment lasted 6 weeks although the number of sessions per week was not reported.	Shallow insertion at acupoints not relevant to the knee.	Manipulated—participants randomised to an acupuncturist who communicated positive messages about acupuncture, for example, “I think this will work for you”, or to neutral communication such as, “It may or may not work for you”.	Self report—pain, satisfaction; physical and mental satisfaction. Objective—range of motion; timed up and go test.

^a^All bilateral acupuncture points stimulated bilaterally unless specified otherwise.

**Table 2:** Summary of included studies' results.

Study	Expectancy	Summary of results^a^
Berk et al. [48]	Manipulated	There were no significant differences between real and placebo acupuncture. There were also no significant differences on shoulder mobility for those given positive versus negative information about acupuncture. Those given positive information reported lower shoulder pain than those given negative information, but this did not reach statistical significance (*P* = 0.053). Interaction between acupuncture and expectancy not reported.

Knox et al. (1979) [52]	Manipulated	There were no significant main effects of acupuncture or expectancy. However, posttreatment experimentally-induced pain reduced significantly from baseline in participants given real acupuncture with positive information but not in participants given real acupuncture with variable or negative information, nor in participants given placebo acupuncture with positive, variable, or negative information.

Norton et al. (1984) [53]	Assessed (dichotomised)	There was a significant interaction between acupuncture and expectancy. Simple effects revealed participants receiving real acupuncture reported significantly less experimentally-induced pain if they had “high expectancy” compared with “low expectancy”. Participants with “high expectancy” who received real acupuncture also reported significantly less pain than those also with “high expectancy” but who received placebo acupuncture. Main effects of acupuncture and expectancy not reported.

Ballegaard et al. (1995) [55]	Assessed (dichotomised)	There were no significant differences on any angina outcome between participants categorised as having “maximal expectancy” and “submaximal expectancy”. Main effect of acupuncture and its interaction with expectancy not reported.

Linde et al. (2007) [49]	Assessed (dichotomised)	Those receiving real acupuncture were more likely to respond to treatment than those receiving placebo acupuncture. Higher expectancies for acupuncture's efficacy in general and specifically for the patients' presenting condition were associated with a higher likelihood of experiencing a 50% improvement in the studies' main outcome and a reduction in pain disability index both immediately posttreatment and at follow up. Significant interaction on “some” outcomes indicating the improved outcomes for those with “high expectancy” compared with “low expectancy” were more marked for patients receiving real acupuncture than those receiving placebo acupuncture.

Bertisch et al. (2009) [50]	Assessed	No significant relationship was found between expectancies and upper arm pain following placebo acupuncture in both unadjusted and multivariate analysis.

Kong et al. (2009) [35, 54]	Manipulated	No main effect of acupuncture. Participants allocated to receive pre-conditioning consistent with acupuncture having an analgesic effect reported significantly less experimentally-induced pain following acupuncture than those allocated to receive pre-conditioning of acupuncture having no effect. There was no interaction between acupuncture and expectancy.

Sherman et al. (2010) [51]	Assessed (trichotomised)	Individualised, standardised, and placebo acupuncture were more effective at reducing chronic low back pain than usual care, but there were no significant differences among these three treatments. There were also no significant differences between those with “high”, “medium”, and “low” expectancies. Interaction between treatment and expectancy not reported.

Suarez-Almazor et al. (2010) [33]	Manipulated	No differences were found between real and placebo acupuncture, but both led to better outcomes compared with the waitlist control group. Participants allocated to receive positive information had significantly lower pain and higher satisfaction than those allocated to receive neutral information and this was independent of whether real or placebo acupuncture was administered.

^a^All results are main effects unless stated otherwise.

**Table 3:** Risk of bias assessment for the included studies.

Study	Adequate sequence generation?	Allocation Concealment?	Blinding?^a^		Incomplete data addressed?	Free of selective reporting bias?	Free of other bias?
			Participant	Outcome Assessor			
Berk et al. (1977) [48]	Unclear	Unclear	Yes	Unclear	Yes	Yes	Yes
Knox et al. (1979) [52]	No	No	Yes	Yes	Yes	Yes	Yes
Norton et al. (1984) [53]	Unclear	Yes	Yes	Unclear	Yes	Yes	No—small sample size for correlational study; dichotomised expectancy
Ballegaard et al. (1995) [55]	Unclear	Unclear	Yes	Yes	Yes	Unclear	No—small sample size for correlational study; dichotomised expectancy
Linde et al. (2007) [49]	Yes	Yes	Yes	Unclear	Yes	Yes	No—dichotomised expectancy
Bertisch et al. (2009) [50]	Yes	Yes	Yes	Yes	Yes	Yes	No—small-medium sample size for correlational study
Kong et al. (2009) [35, 54]	Unclear	Unclear	Yes	Unclear	Yes	Yes	Yes
Sherman et al. (2010) [51]	Yes	Yes	Yes	Yes	Yes	Yes	No—trichotomised expectancy
Suarez-Almazor et al. (2010) [33]	Yes	Yes	Yes	Yes	Yes	Yes	Yes

^a^Risk of bias for blinding was assessed only for whether participants were intended to be blind to the type of acupuncture they received (real or placebo) and whether outcome assessors were blind to the participants' allocation. Blinding of acupuncturists regarding acupuncture treatment is not possible, nor is it possible to blind participants regarding an expectancy manipulation; therefore, these were not included in the risk of bias assessment. ^b^In Bertisch et al. [50], even though only placebo acupuncture was delivered for the period of interest, they were told they may receive real or placebo acupuncture and are, therefore, considered as blind to treatment allocation.
